# Potential Mechanisms Involved in the Protective Effect of Dicaffeoylquinic Acids from *Artemisia annua* L. Leaves against Diabetes and Its Complications

**DOI:** 10.3390/molecules27030857

**Published:** 2022-01-27

**Authors:** Hesham El-Askary, Heba H. Salem, Amira Abdel Motaal

**Affiliations:** 1 Department of Pharmacognosy, Faculty of Pharmacy, Cairo University, Cairo 11562, Egypt; aabdulmtaal@kku.edu.sa; 2 Department of Biochemistry, Faculty of Pharmacy, Cairo University, Cairo 11562, Egypt; 3 College of Pharmacy, King Khalid University, Abha 61441, Saudi Arabia

**Keywords:** diabetes, DPPIV, α-glucosidase, α-amylase, aldose reductase, antioxidant, wound healing, diabetes complications, *Artemisia annua*, dicaffeoylquinic acids

## Abstract

Diabetes mellitus is a chronic disease affecting the globe and its incidence is increasing pandemically. The use of plant-derived natural products for diabetes management is of great interest. Polar fraction of *Artemisia annua* L. leaves has shown antidiabetic activity in vivo. In the present study, three major compounds were isolated from this polar fraction; namely, 3,5-dicaffeoylquinic acid (**1**); 4,5-dicaffeoylquinic acid (**2**), and 3,4- dicaffeoylquinic acid methyl ester (**3**), using VLC-RP-18 and HPLC techniques. The potential protective effects of these compounds against diabetes and its complications were investigated by employing various in vitro enzyme inhibition assays. Furthermore, their antioxidant and wound healing effectiveness were evaluated. Results declared that these dicaffeoylquinic acids greatly inhibited DPPIV enzyme while moderately inhibited α-glucosidase enzyme, where compounds **1** and **3** displayed the most prominent effects. In addition, compound **3** showed pronounced inhibition of α-amylase enzyme. Moreover, these compounds markedly inhibited aldose reductase enzyme and exerted powerful antioxidant effects, among which compound **3** exhibited the highest activity implying a notable potentiality in impeding diabetes complications. Interestingly, compounds **2** and **3** moderately accelerated scratch wound healing. Our findings suggest that these dicaffeoylquinic acids can be promising therapeutic agents for managing diabetes and its complications.

## 1. Introduction

Diabetes mellitus is a chronic metabolic disorder defined by elevated blood glucose level due to defective insulin secretion [1]. This disease is a cardinal global health condition, and its incidence is increasing progressively in a pandemic way. Despite the presence of high number of marketed pharmaceuticals for diabetes treatment [2], there is still a lack of satisfactory effective and safe therapy with the existing agents. In recent years, there has been an expanded interest in the use of therapeutically active herbal products for diabetes management due to their low cost, higher accessibility and relative safety [3,4].

Interestingly, several species of *Artemisia* are used traditionally in different countries for the treatment of diabetes and their extracts have shown antidiabetic activities in different in vivo animal models of diabetes [5]. *Artemisia annua* L., family Asteraceae is an annual herb native to China and is known traditionally for treating malaria, and as a source of artemisinin. This plant started to attract much attention due to its wide spectrum of biological activities including antimalarial, antimicrobial, anti-inflammatory, antioxidant, immunomodulatory and anticancer activities [6,7]. Aqueous extract of *A. annua* was reported to reduce glucose level and to increase insulin level in serum of diabetic rats [8]. Previous work done by members of our group showed that administration of the polar fraction from the leaves of *A. annua* (ART-CQ) cultivated in Egypt effectively lowered blood glucose level and normalized blood glutathione level in alloxan-induced diabetic rats [9]. An HPLC/PDA/ESI/MS-MS-based analysis carried out for the ART-CQ revealed the presence of several quinic acid derivatives [10] and some of these derivatives were isolated [9]. However, the different mechanisms of action behind the antidiabetic activity of ART-CQ were never explored; also, the major biologically active compounds responsible for this activity were not detected.

Various plant products exert their antidiabetic effects through inhibiting the activity of several enzymes critical for diabetes management, including α-glucosidase, α-amylase, dipeptidyl peptidase IV (DPP IV) and aldose reductase [11,12]. Incretins are intestinal hormones that modulate blood glucose level by stimulating insulin secretion while inhibiting glucagon release [13]. In addition, they increase the survival and inhibit apoptosis of pancreatic β-cells. Hence, a new promising class of antihyperglycemic agents has emerged that act by inhibiting DPPIV enzyme, an enzyme responsible for the inactivation of the incretins [14]. Furthermore, the antidiabetic potential of many herbal products was attributed to the inhibition of this enzyme [15]. The intestinal enzymes, α-glucosidase and α-amylase are essential for glucose absorption via the hydrolysis of dietary carbohydrates and as such they are significant contributors to postprandial hyperglycemia. Accordingly, targeting these enzymes plays an important role in diabetes management [16,17].

It has been well known that diabetes is accompanied by the occurrence of multiple microvascular and macrovascular complications [18]. Increased blood glucose level leads to enhanced flux through the polyol pathway with the resultant accumulation of sorbitol that is implicated in the progression of many vascular and neurological complications of diabetes [18]. Aldose reductase is the rate limiting enzyme in this pathway; therefore, inhibiting this enzyme has a potential protective impact against diabetes complications [19]. It is worth mentioning that diabetes development has been proven to be associated with a state of increased oxidative stress that is crucially involved in the pathogenesis of diabetes complications [20,21]. Thus, the use of antioxidants is of considerable value in ameliorating diabetes complications [22]. One of the common complications of diabetes is impaired wound healing [23]. This can be attributed to hyperglycemia, inflammation and micro and macrovascular dysfunction that can give rise to chronic ulcers formation including diabetic foot ulcers. In spite of the presence of various treatments for chronic wounds, only few are effective which necessitates the development of alternative safe remedies using natural products [24].

Considering the aforementioned reports and driven by our interest in *A. annua*, the present study aimed at the isolation and identification of the major compounds present in ART-CQ and exploring their biological activity as antidiabetic lead compounds through different mechanisms of action. This was accomplished by the determination of their in vitro inhibitory effectiveness against various enzymes. Furthermore, the in vitro antioxidant and wound healing potential of ART-CQ and the isolated major compounds was also evaluated as potential protective mechanisms against diabetes complications.

## 2. Results and Discussion

### 2.1. HPLC/RP-18 Fingerprint Chromatogram of ART-CQ

**Figure 1:**
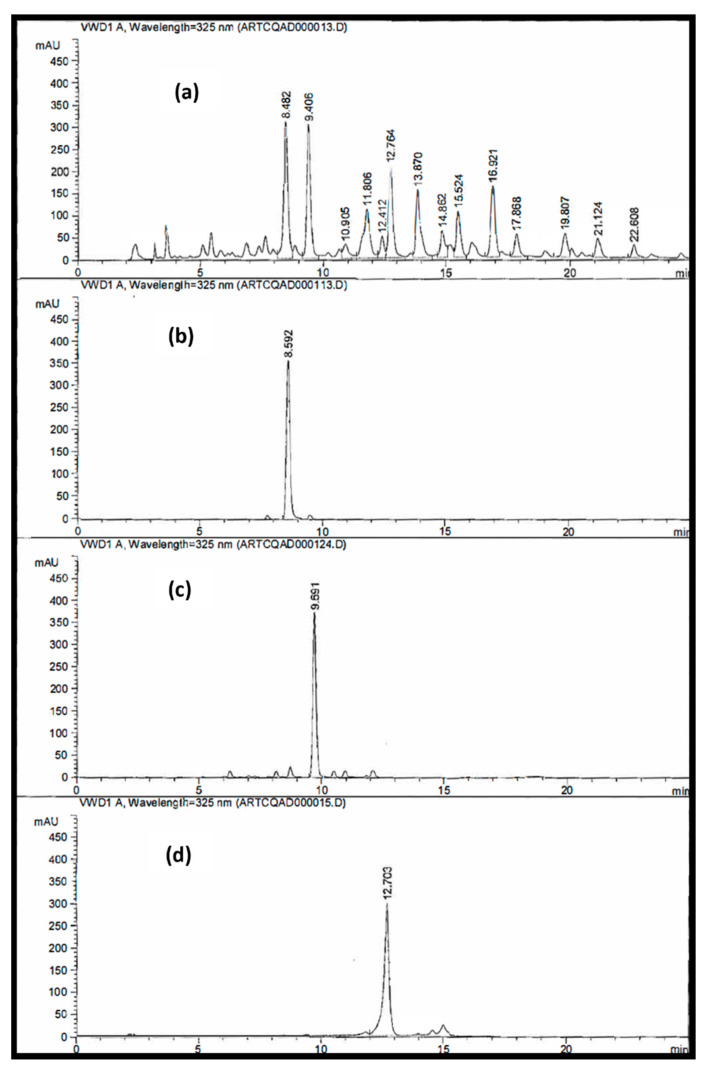
HPLC/RP-18 fingerprint chromatographic profile. (**a**), biologically active polar fraction of *Artemisia annua*, ART-CQ; (**b**), 3,5-dicaffeoylquinic acid (**1**) at Rt 8.5 min; (**c**), 4,5-dicaffeoylquinic acid (**2**) at Rt 9.5 min; (**d**), 3,4-dicaffeoylquinic acid methyl ester (**3**) at Rt 12.7 min.

HPLC chromatogram of the combined polar fraction of *A. annua* (ART-CQ, 3 mg/3 mL M/W) showed several peaks for quinic acid derivatives (Figure 1). Out of these, three major peaks were detected (at Rt 8.5, 9.5 and 12.7 min) and were subjected to chromatographic separation, using VLC-RP-18 column, monitored by HPLC analysis.

### 2.2. Identification of the Three Isolated Compounds

**Figure 2:**
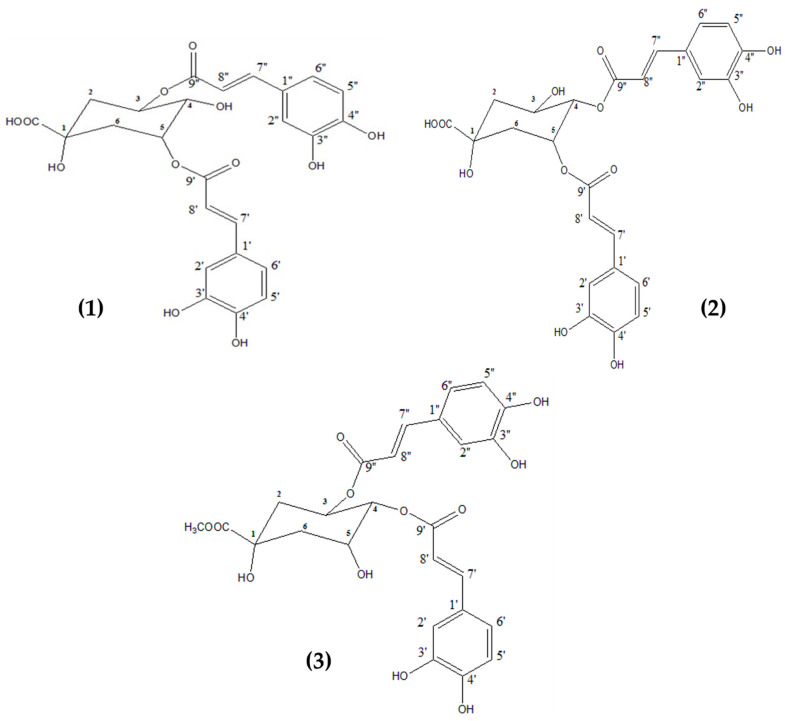
Chemical structure of the isolated compounds from the polar fraction of *Artemisia annua*. 3,5-dicaffeoylquinic acid (**1**); 4,5-dicaffeoylquinic acid (**2**); 3,4-dicaffeoylquinic acid methyl ester (**3**).

Three major compounds (**1**–**3**) were isolated from the polar fraction ART-CQ using different chromatographic techniques. Based on their spectroscopic data; ^1^H NMR and co-chromatography with previously isolated compounds, these compounds were identified as 3,5-dicaffeoylquinic acid (**1**); 4,5-dicaffeoylquinic acid (**2**) [9,25], and 3,4-dicaffeoylquinic acid methyl ester (**3**) [9,26] (Figure 2).

### 2.3. Dipeptidyl Peptidase IV (DPPIV) Inhibition

**Figure 3:**
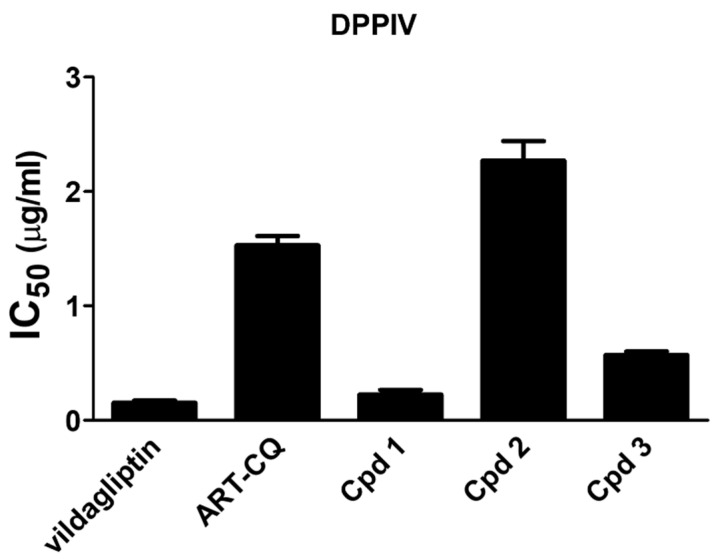
Inhibition of Dipeptidyl Peptidase IV (DPPIV) enzyme by the combined polar fraction of *Artemisia annua* (ART-CQ) and the isolated compounds (**1**–**3**). Values are shown as mean ± SEM of two replicates. Cpd, compound.

Incretins are gut hormones that play an important role in glucose homeostasis by stimulating glucose-dependent insulin secretion, together with their anti-apoptotic and proliferation-enhancement effects on pancreatic β-cells [13]. Therefore, as these hormones are inactivated by dipeptidyl peptidase IV (DPPIV) enzyme, inhibition of this enzyme has emerged as a promising approach for controlling blood glucose [14,27]. Based on these facts, the in vitro DPPIV inhibition capability of the polar fraction of *A. annua* extract (ART-CQ) and its isolated compounds was assessed as a potential antidiabetic mechanism. Interestingly, as shown in Figure 3 the ART-CQ and the isolated compounds **1**, **2**, **3** have displayed potent DPPIV inhibitory activities with IC_50_ values of 1.53 ± 0.08, 0.225 ± 0.04, 2.27 ± 0.17, 0.571 ± 0.03 µg/mL, respectively. Notably, compound **1** approached the activity of vildagliptin (IC_50_ 0.154 ± 0.02 µg/mL).

### 2.4. Intestinal Enzymes Inhibition

The intestinal enzymes, α-amylase and α-glucosidase, play an essential role in the hydrolysis of polysaccharides and disaccharides into glucose. Targeting these enzymes is one of the current strategies for diabetes management [16]. Thus, the effects of ART-CQ and its isolated compounds on the α-amylase and α-glucosidase activities were measured in the present study.

**Figure 4:**
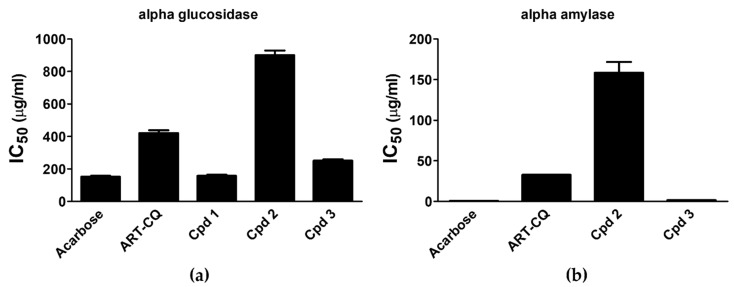
Alpha glucosidase (**a**) and α-amylase (**b**) inhibitory activities of the combined polar fraction of *Artemisia annua* (ART-CQ) and the isolated compounds (**1**–**3**). Values are shown as mean ± SEM of two replicates. Cpd, compound.

Results indicated that compound **1** possessed considerable α-glucosidase inhibitory activity (IC_50_ 157.13 ± 6.82 µg/mL) which was similar to the standard acarbose (IC_50_ 151.97 ± 5.85 µg/mL) (Figure 4a). Meanwhile, compound **3** possessed moderate α-glucosidase inhibitory activity (IC_50_ 250.42 ± 8.44 µg/mL) while the extract and compound **2** showed weak activities (IC_50_ 421.36 ± 16.1, 900.04 ± 29.2, respectively) (Figure 4a). As for the α-amylase inhibition assay, results illustrated in Figure 4b revealed that compound **3** induced the highest inhibition of the enzyme activity with IC_50_ value of 1.51 ± 0.01 µg/mL that was comparable to the standard acarbose value (0.57 ± 0.04 µg/mL). In addition, ART-CQ and compound **2** exerted efficient reduction of α-amylase activity (IC_50_ 32.72 ± 0.13, 158.45 ± 13.25, respectively) (Figure 4b). However, compound **1** had no α-amylase inhibitory effect.

### 2.5. Aldose Reductase Inhibition

**Figure 5:**
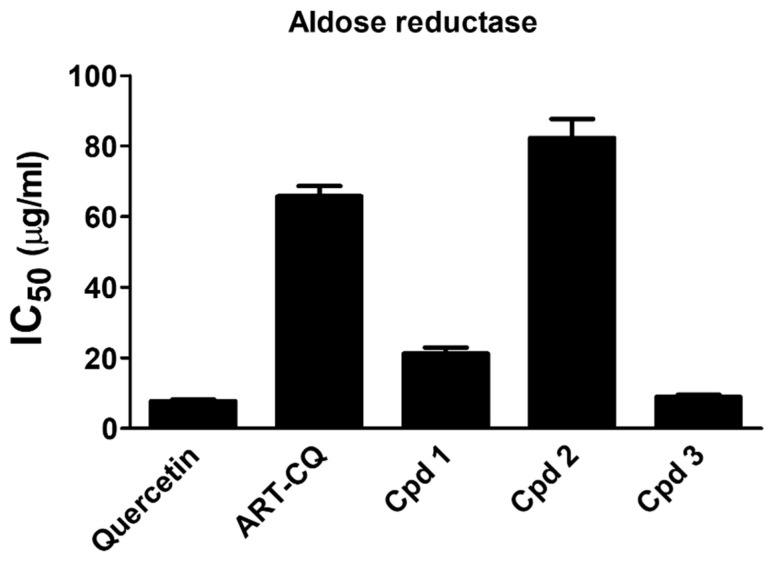
Inhibition of aldose reductase enzyme by the combined polar fraction of *Artemisia annua* (ART-CQ) and the isolated compounds (**1**–**3**). Values are shown as mean ± SEM of two replicates. Cpd, compound.

Increased activity of aldose reductase is associated with the development of different diabetes complications such as cataract, neuropathy, nephropathy and cardiovascular complications. Therefore, approaches aiming at repressing this enzyme are of great value in managing diabetes complications [19]. In this study, we have investigated the in vitro aldose reductase inhibition potential of the combined polar fraction of *A. annua* extract and its isolated compounds. Results depicted in Figure 5 unveiled that the ART-CQ and the isolated compounds have displayed excellent aldose reductase inhibitory activities. Remarkably, compound **3** exhibited the most prominent activity (IC_50_ 8.90 ± 0.61 µg/mL) which was comparable to the standard quercetin (IC_50_ 7.77 ± 0.43 µg/mL). Moreover, the ART-CQ and compounds **1** and **2** had IC_50_ values of 65.74 ± 2.91, 21.22 ± 1.66 and 82.25 ± 5.41, respectively.

Various studies have reported that dicaffeoylquinic acids exerted antihyperglycemic effect and preserved pancreatic cell function and structure in various models of diabetes [28,29,30]. Indeed, the aforementioned results proved that dicaffeoylquinic acids might contribute to the antidiabetic effect of *A. annua* polar fraction through various mechanisms. Intriguingly, the three dicaffeoylquinic acid derivatives greatly inhibited the DPPIV enzyme activity, an effect that could potentially convey the antidiabetic effect of *A. annua*. DPPIV enzyme inhibitors have emerged as efficient antidiabetic medications that are already available in the market and their usage has expanded markedly with the advantages of low risk of hyperglycemia and being orally active [31]. Inhibition of DPPIV enzyme could modulate hyperglycemia by increasing endogenous incretin hormones level with the resultant increment in insulin secretion and enhancement of pancreatic β-cell survival [13]. Our findings might thus explain the reported increase in plasma insulin level and pancreatic islet mass by the administration of a butanol fraction of *Cichorium glandulosum* seeds, which was rich in dicaffeoylquinic acids, to streptozotocin-induced diabetic mice with the consequent improvement in glucose tolerance [28]. This fraction contained 3,5-dicaffeoylquinic acid as the major component in addition to the presence of moderate amounts of 3,4- and 4,5-dicaffeoylquinic acids. Consistently, a methanolic extract of *Gynura divaricata* enriched in 3,5- and 4,5-dicaffeoylquinic acids decreased blood glucose level and pancreatic apoptosis while enhanced β-cell regeneration in diabetic mice [32].

Moreover, our data implied that the three dicaffeoylquinic acid derivatives could also mediate the antidiabetic potential of *A. annua* polar fraction via inhibiting the α-glucosidase enzyme, thus impeding glucose absorption and preventing postprandial hyperglycemia. Notably, compound **1** displayed the highest α-glucosidase inhibitory activity in accordance with previous literatures illustrating very potent α-glucosidase inhibitory activities of 3,5-dicaffeoylquinic acid from *Cichorium glandulosum* [28] and from *Geigeria alata* to which its antihyperglycemic effect was attributed [30]. Our results are in agreement as well with the reported α-glucosidase inhibitory activity of 4,5-dicaffeoylquinic acid and 3,4-dicaffeoylquinic acid methyl ester (compound **2** and **3**) from *Gynura divaricata* [33]. Furthermore, other compounds were isolated for the leaves and stems of *A. annua* that showed α-glucosidase inhibitory effectiveness [34]. This, together with our finding, would suggest that inhibition of α-glucosidase enzyme might play a significant role in the antidiabetic activity of *A. annua*. On the other hand, compound **3** exhibited a substantial α-amylase inhibition activity while compound **2** showed moderate activity. This is in accordance with the previously described α-amylase inhibitory activity for 4,5-dicaffeoylquinic acid [35,36]. Interestingly, molecular docking has illustrated that dicaffeoylquinic acid exhibited the most fitting for the active sites of α-glucosidase and α-amylase enzymes among the major compounds isolated from *Chasmanthe aethiopica* extract which justified its antihyperglycemic and insulin increasing effects [37].

Intriguingly, the combined polar fraction of *A. annua* together with its three major biologically active dicaffeoylquinic acid derivatives potentially offer protection against diabetes complications by efficiently inhibiting aldose reductase enzyme activity. Our findings were in harmony with the study by Kuroda and his colleagues who have reported a marked aldose reductase inhibitory activity by the same dicaffeoylquinic acid derivatives isolated from the leaves of *Tussilago farfara* [38]. In addition, the same study demonstrated that the methanolic extract of different species of *Artemisia*; *A. absinthium, A. dracunculus* and *A. vulgaris* exerted potent inhibition of aldose reductase enzyme. Moreover, 4,5-dicaffeoylquinic acid isolated from an antidiabetic methanolic extract of *A. dracunculus* showed good aldose reductase inhibitory efficacy [39].

### 2.6. In Vitro Antioxidant Activity

**Figure 6:**
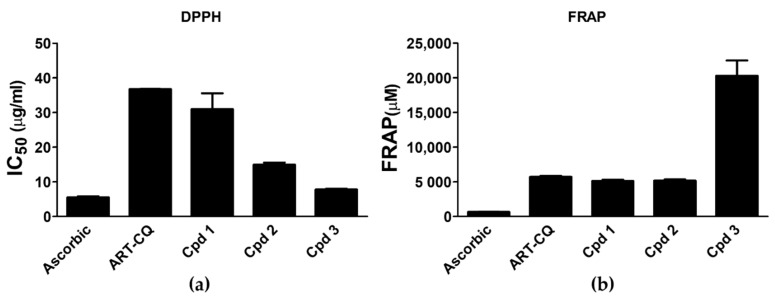
Antioxidant activity of the combined polar fraction (ART-CQ) and isolated compounds (**1**–**3**) from *Artemisia annua*. (**a**) DPPH radical scavenging. (**b**) Ferric reducing antioxidant power (FRAP). Values are shown as mean ± SEM of two replicates. Cpd, compound; DPPH, 2,2-diphenyl-1-picrylhydrazyl.

One of the important molecular mechanisms implicated in the development of diabetes complications is enhanced oxidative stress [20] and the use of antioxidants is considered as one of the useful measures for protecting against these complications [22]. Based on the abovementioned facts, the antioxidant and free radical scavenging potential of the tested samples was assessed using the Ferric Reducing Antioxidant Power (FRAP) and 2, 2-Diphenyl-1-picrylhydrazil (DPPH) assays. Results of the DPPH assay declared that the ART-CQ and its isolated compounds (**1**–**3**) possessed potent free radical scavenging capability where 50% scavenging was attained at concentrations (IC_50_) of 36.75 ± 0.015, 30.94 ± 4.61, 14.94 ± 0.60 and 7.78 ± 0.24 µg/mL, respectively (Figure 6a). Compound **3** demonstrated the highest activity that was comparable to the reference ascorbic acid (IC_50_ 5.50 ± 0.30 µg/mL) (Figure 6a). Amazingly, on the other hand, all samples showed powerful antioxidant activity in the FRAP assay that greatly surpassed that of the ascorbic acid positive control where the FRAP values of the ART-CQ and compounds (**1**,**2**) were about 8 times that of the ascorbic acid (Figure 6b). In agreement with the DPPH results, compound **3** had the most prominent antioxidant activity with tremendous FRAP value of more than 30 folds of that of ascorbic acid (Figure 6b).

Our findings are in agreement with the previously shown in vitro [10] and in vivo [9] antioxidant activity of the ART-CQ. The polar fraction of *A. Annua* greatly scavenges the 2,2′-azino-bis (3-ethylbenzothiazoline-6-sulphonic acid) (ABTS) free radicles in vitro [10] and normalized blood glutathione level of alloxan diabetic rats [9]. Furthermore, these data are in line with the previously reported potent antioxidant activities of 3,5- and 4,5-dicaffeoylquinic acids that were delineated as the major antioxidant metabolites isolated from different herbs of family Asteraceae [40] and also from *Scabiosa comosa* and *S. tschilliensis* [41]. The combination of the catechol group and three unsaturated ester moieties in the structure of compound **3** (3,4- dicaffeoylquinic acid methyl ester) accounted for the marked increase in its antioxidant activity [42]. In solidarity, in vivo administration of 3,5-dicaffeoylquinic acid in streptozotocin-induced diabetic rats reduced blood glucose level and displayed antioxidant activity where it reduced malondialdehyde level and enhanced glutathione level and the endogenous antioxidant enzymes activities [29,30]. Treatment of diabetic mice with dicaffeoylquinic acids-enriched extract of *Gynura divaricata* increased glutathione peroxidase and superoxide dismutase activities while mitigating malondialdehyde level [32]. The impressive antioxidant properties of *A. annua* polar fraction and its dicaffeoylquinic acid derivatives enable them to be potential candidates for protection against diabetes complications by combating the hyperglycemia-provoked oxidative stress.

### 2.7. In Vitro Cell Viability Cytotoxicity

Cell viability assay was conducted to evaluate the effect of the ART-CQ and its major biologically active compounds on the viability of human skin fibroblast (HSF) cells and to select the appropriate concentrations to be used in the wound healing assay. Incubating the HSF cells with the ART-CQ and the isolated compounds for 72 h had no significant cytotoxic effect and more than 80% of cells remain viable up to the upper concentration, 100 µg/mL. The IC_50_ values were calculated as 1905 µg/mL, 3393 µg/mL, 3577 µg/mL and 8575 µg/mL for the combined polar fraction (ART-CQ), compound **1**, **2** and **3**, respectively. This indicated the high safety of the polar fraction of the *A. annua* and its major isolated compounds.

### 2.8. In Vitro Wound Healing

**Figure 7:**
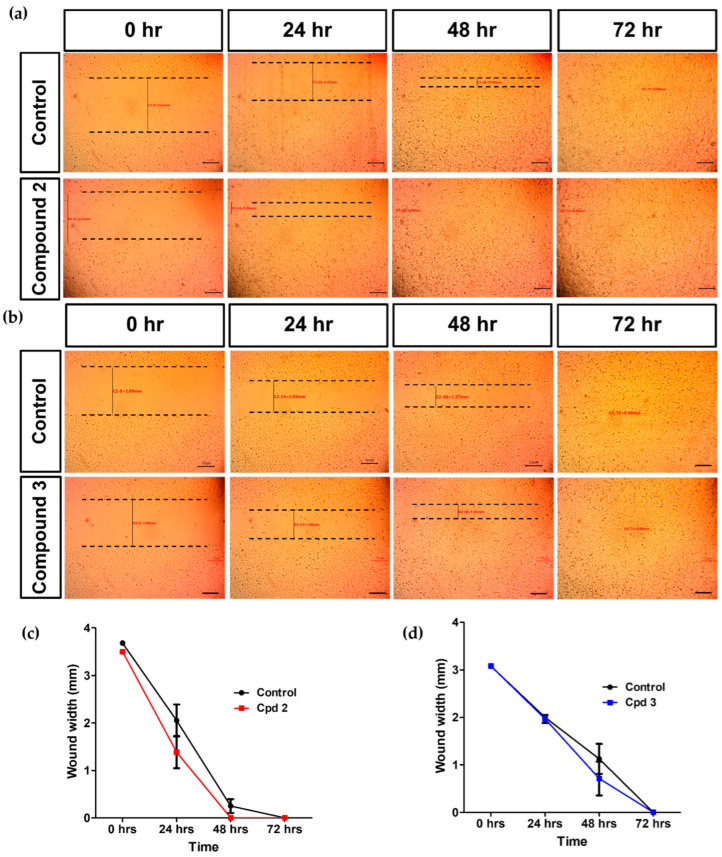
Effect of compound **2** and **3** on wound healing. Scratch assay images of human skin fibroblast (HSF) cells treated with 10 µg/mL of compound **2** (**a**) and compound **3** (**b**) at different time points as compared to the control. Wound width of HSF cells treated with 10 µg/mL of compound **2** (**c**) and compound **3** (**d**) at different time points as compared to the control. Experiments were done in triplicates.

Diabetes mellitus is accompanied by defective wound healing that can lead to one of the serious complications of diabetes, the diabetic foot ulcer [23]. In the current study, we examined the effect of ART-CQ and its major isolated compounds on wound healing in layers of HSF cell lines using the in vitro scratch wound healing model. Results declared that compounds **2** and **3** improved the wound healing at a concentration of 10 µg/mL. Treating the HSF with compound **2** enhanced cell migration and accelerated the scratch wound healing where wound width being reduced to 40% of the original wound width as compared to only 56% in the control at 24 h (Figure 7a,c). Furthermore, wounds were completely healed by in 48 h while wounds in the control plates were healed only in 72 h (Figure 7a,c). Compound **3** showed increased cell migration only at 48 h with decreased wound width to 23% compared to 37% in the control and both wounds were healed completely in 72 h (Figure 7b,d). However, the combined polar fraction of *A. annua* did not accelerate wound healing at any of the tested concentrations while compound **1** inhibited cell migration and wound healing at all tested concentrations.

Cell migration is one of the important events driving wound closure and healing [24]. Compound **2**, and to a lesser extent compound **3**, showed enhanced cell migration suggesting a potential efficacy in diabetic wound healing. A methanolic extract of *Artemisia annua* was used for the first time for the composition of a wound dressing that was shown to increase proliferation of L929 fibroblast cells but only after 4 days of incubation and to exert potent antibacterial activity [43]. Furthermore, accelerated wound healing was described in other *Artemisia* species [44,45]. Rhimi et al. (2019) has reported enhanced wound healing in mice by using an ointment of the ethanolic extract of *Dittrichia viscosa*, family Asteraceae [46]. For our concern, this extract was enriched with different dicaffeoylquinic acid derivatives including 4,5- and 3,4-dicaffeoylquinic acids. In line with our elucidated wound healing promoting effect of compound **2** (4,5-dicaffeoylquinic acid), Kurisu and his coworkers demonstrated the induction of the mitogenic factor hepatocyte growth factor by this compound in Neonatal Normal Human Dermal Fibroblasts [47]. Additionally, 4,5-dicaffeoylquinic acid from *Artemisia absinthium* potentiated the effect of antibacterial agents against bacteria that could potentially infect the wounds as *Straphylococcus aureus* [48]. Taken together, 4,5-dicaffeoylquinic acid (compound **2**) could be a promising agent that might promote diabetic wound healing.

## 3. Materials and Methods

### 3.1. Plant Material

*A. annua* leaves were obtained from the Experimental Station of Medicinal Plants, Faculty of Pharmacy, Cairo University in July 2012. This time was selected as this is the season where the highest content of quinic acid derivatives can be obtained at the pre-flowering stage of the plant, which is the same stage where the artemisinin content is the highest [9]. The plant was authenticated by Prof. Dr. Ibrahim El-Garf, Professor of Botany, Department of Science, Cairo University. A voucher specimen (13 April 2014) was deposited at the herbarium at the Pharmacognosy Department, Faculty of Pharmacy, Cairo University, Egypt.

### 3.2. General

Alpha-amylase (porcine pancreas), 2-chloro-4-nitrophenyl-D-maltotrioside (CNPG3) and tetramethylsilane were from Sigma-Aldrich (St. Louis, MO, USA) while the remaining chemicals were from UFC Biotechnology. Spectrophotometric readings were performed using the microplate reader (Anthos Zenyth 200RT, Cambridge, UK). ^1^H-NMR (400 MHz) analysis was carried out using Bruker NMR Spectrometer (Bruker, Yokohama, Japan). Spectra were recorded in CD_3_OD or DMSO-d_6_ with tetramethylsilane being employed as an internal standard, and chemical shift values were expressed in ppm.

### 3.3. Extract Preparation

The air-dried leaves of *A. annua* (1 kg) were extracted with 70% ethanol by sonication till exhaustion, yielding a green residue (240 g). The alcoholic extract (200 g) was extracted successively with hexane, chloroform, ethyl acetate and n-butanol [10]. The combined polar fraction (ART-CQ, 49 g) was used for the current study.

### 3.4. HPLC/RP-18 Analysis of ART-CQ

HPLC apparatus: Agilent Technologies 1100 series, HPLC system (Agilent Technologies, Palo Alto, CA, USA), equipped with a quaternary pump and degasser G1322A series 1200 was used. Agilent Chem Station software was used for data acquisition and processing [9].

HPLC conditions: HPLC analysis was carried out on Lichro-Chart (250 × 4 mm) Lichrosphere RP-18 (5 µm) Merck column, together with a Guard column (10 × 4 mm, 5 µm). Mobile phase: Acetonitrile “solvent A” and 0.3% H_3_PO_4_ in H_2_O “solvent B” applying gradient elution: 20% A/B to 45% A/B in 25 min, then to 100% A in 2 min. and return back to 20% A/B in 2 min. Flow rate 1 mL/min, injection volume 20 µL, and UV detection at 325 nm.

### 3.5. Isolation of the Major Compounds from ART-CQ

Two parts of the polar fraction (ART-CQ) each of 1.2 g was dissolved in 10% methanol/water (M/W), applied on VLC-RP-18 column (RP-18 silica, 42 gm, 5.5 cm L × 4 cm D), and elution was started with 10% M/W, with gradual increase of methanol in water till 25% M/W. Fractions of 100 mL each, were collected and monitored by HPLC to give three major collective fractions; I-III. Collective fraction I (eluted with 10–12% M/W, 600 mL), showed major peak (65% purity) at Rt 8.5 min. It was further purified using VLC-RP-18 column giving compound (**1**) (74 mg). Collective fraction II (eluted with 14–16% M/W, 500 mL), showed major peak (68% purity) at Rt 9.5 min, which was purified by repeated VLC-RP-18 columns giving compound (**2**) (62 mg). While collective fraction III (eluted with 19–22% M/W, 700 mL), showed major peak (54% purity) at Rt 12.7 min, and was further purified on VLC-RP-18 columns giving compound (**3**) (38 mg).

### 3.6. Spectroscopic Data of the Isolated Compounds

Compound (**1**)

UV spectral data (MeOH) λ_max_ = 244–327 nm. ^1^H NMR, δ ppm (400 MHz. DMSO-d_6_): quinic acid moiety; 2.1 (2H, m. H-6 axe, eq), 2.50 (2H, m, H-2 axe, eq), 3.77 (lH, m, H-4). 5.2, 5.25 (2H, m. H-5. H-3). Two caffeic acid moieties; 6.34 (lH, d, J = 15.9 Hz. H-8″), 6.29 (lH, d, J = 15.9 Hz, HS′), 6.79 (2H, d, J = 7 Hz, H-5″, 5′), 6.98 (2H, m. H6″, 6′), 7.03 (2H, brs, H-2″. 2′), 7.56 (lH, d, J = 15.9 Hz, H-7″), 7.6 (lH, d, J = 15.9 Hz, H-7′).

Compound (**2**)

UV spectral data (MeOH) λ_max_ = 245–327 nm. ^1^H NMR δ ppm (400 MHz, DMSO-d_6_): quinic acid moiety; 2.13 (lH, m. H-6 axe), 2.17 (2H. m. H-2 axe, eq), 2.28 (lH, m. H-6 eq), 4.36 (lH, m, H-3), 5.11 (lH, m, H-4), 5.62 (lH, m, H-5). Two caffeic acid moieties; 6.20 (lH, d, J = 16 Hz. H-8″), 6.31 (lH, d, J = 16 Hz, H-8′), 6.75 (2H. d, J = 8.1 Hz, H-5″,5′), 6.92 (2H. m, H-6″,6′), 7.04 (lH, brs, H2″,2′), 7.46 (lH, d, J = 16 Hz, H-7″), 7.61 (lH, d, J = 16.2 Hz. H-7′).

Compound (**3**)

UV spectral data (MeOH) λ_max_ = 244–327 nm. ^1^H NMR δ ppm (400 MHz. DMSO-d_6_): quinic acid moiety; 1.8 (lH, m, H-6 ax, eq), 2.09 (2H, m. H2-2 ax, eq), 2.17 (lH, m, H-6 eq), 3.73 (3H, s, OCH3), 4.2 (lH. m. H-5), 4.88 (lH, m, H-4), 5.55 (lH, m, H-3). Two caffeic acid moieties; 6.18 (lH. d,J = 15 Hz, H-8″), 6.36 (lH. D, J = 15 Hz, H-8′), 6.72 (2H, d,J = 8.1 Hz, H-5″,5′), 6.96 (2H, m, H-6″,6′), 7.01 (2H. brs, H2″, 2′), 7.42 (lH, d, J = 16 Hz. H-7″), 7.48 (lH. d, J = 16.2 Hz. H-7′).

### 3.7. In Vitro Antidiabetic Activity

#### 3.7.1. Dipeptidyl Peptidase IV (DPPIV) Inhibition Assay

The dipeptidyl peptidase IV (DPPIV) inhibition effectiveness of the polar fraction of *Artemisia annua* (ART-CQ) and the isolated compounds was determined fluorometrically [49] by using the DPPIV Inhibitor Screening Kit (Abnova, Taipei, Taiwan) following the manufacturer’s protocol. Briefly, different concentrations of either ART-CQ (10, 50, 100, 200 µg/mL) or the pure compounds (5, 20, 50, 100 µg/mL) were preincubated in a plate with the enzyme solution at 37 °C for 10 min. Then the enzyme substrate solution was added, and the absorbance was measured at Ex/Em = 360/460 nm. The plate was reincubated for 30 min at 37 °C and the absorbance was recorded again. Vidagliptin was used as the reference standard. The difference between the two readings was estimated and the IC_50_ was determined.

#### 3.7.2. α-Glucosidase Inhibition Assay

The in vitro α-glucosidase inhibitory potential of ART-CQ and its isolated compounds was evaluated using the α-Glucosidase Inhibitor Screening Kit (Biovision, Milpitas, CA, USA). In a 96 well plate, 10 µL of the test samples and the standard acarbose was incubated with 10 µL Diluted Enzyme Solution for 20 min at room temperature. After incubation, 20 µL of the α-Glucosidase Substrate in buffer was added and the absorbance was measured kinetically at 410 nm for 60 min. The polar fraction was tested in concentrations (10, 50, 100, 200 µg/mL) while the concentrations of the isolated compounds were (5, 20, 50, 100 µg/mL). Acarbose was the standard used in the assay and an enzyme control was carried out in which the samples were replaced by the assay buffer.

The change in absorbance is directly proportional to enzyme activity and the presence of enzyme inhibitor reduces the formation of the end product with the resultant decrease in absorbance. The concentration required to inhibit 50% of enzyme activity, IC_50_ for test compounds as well as the standard was determined.

#### 3.7.3. α-Amylase Inhibition Assay

The inhibitory effect of the polar fraction (ART-CQ) and isolated compounds from *Artemisia Annua* on the activity of α-amylase enzyme was assessed kinetically as previously described [27]. This assay depends on the hydrolysis of 2-chloro-4-nitrophenyl maltotrioside (CNPG3) by the α-amylase enzyme with the production of 2-chloro-4-nitrophenol that can be measured spectrophotometrically at 405 nm for 3 min. The change in absorbance per min is proportional to the enzyme activity. Various concentrations of ART-CQ (10, 50, 100, 200 µg/mL) and isolated compounds (5, 20, 50, 100 µg/mL) were preincubated with the enzyme for 15 min at 37 °C before the addition of CNPG3. A standard acarbose and a sample-free control were used in the assay and the 50% inhibitory concentrations (IC_50_) were calculated for all tested samples and the standard.

#### 3.7.4. Aldose Reductase Inhibition Assay

Aldose reductase inhibitory activity was determined kinetically by measuring the decrease in the absorption of NADPH at 340 nm in a reaction mixture containing, in addition to NADPH, the enzyme, DL-glyceraldehyde and the samples [50]. This was accomplished using the Aldose Reductase Inhibitor Screening Kit (Biovision, Milpitas, CA, USA), in accordance with the manufacturer’s instructions. In brief, 10 μL of either the Enzyme Assay Buffer, ART-CQ (10, 50, 100, 200 µg/mL) or the isolated compounds (5, 20, 50, 100 µg/mL) were placed in a 96 well plate. Then 60 μL of NADPH and 90 μL of enzyme solution were added and the plate was incubated in dark for 20 min at 37 °C. Subsequently, 40 μL of DL-glyceraldehyde was pipetted and the absorbance was determined at 340 nm at 37 °C for 90 min. Quercetin was utilized as the standard and the IC_50_ for the investigated samples and the standard was calculated.

### 3.8. In Vitro Antioxidant Activity

#### 3.8.1. Ferric Reducing Antioxidant Power (FRAP) Assay

The DetectX^®^ Ferric Reducing Antioxidant Power (FRAP™) Detection Kit (Arbor assays, Ann Arbor, MI, USA) was used to estimate the antioxidant and ferric reducing activity of the polar fraction of *A. annua* and the isolated compounds in accordance with the method of Benzie and Strain (1996) [51]. ART-CQ was utilized in a concentration of (50 µg/mL) while the isolated compounds were used in a concentration of (25 µg/mL). Ferric chloride standard and ascorbic acid control were used in the assay and the FRAP values were estimated and presented as µM FeCl_2_.

#### 3.8.2. 2,2-Diphenyl-1-picrylhydrazil (DPPH) Radical Scavenging Assay

2,2-diphenyl-1-picrylhydrazil (DPPH) Radical Scavenging Assay was exploited to assess the free radical scavenging potential of ART-CQ and the pure compounds according to Motaal et al., 2011 [52]. Different concentrations of ART-CQ (8, 16, 48, 64 µg/mL) and the isolated compounds (2, 4, 12, 16 µg/mL) were used. Ascorbic acid was used as the standard positive control and water was used in place of samples as a blank. The IC_50_ value for the test samples and standard was evaluated.

### 3.9. In Vitro Cell Culture

Human Skin Fibroblasts (HSF) cell line was obtained from Nawah Scientific Inc. (Mokatam, Cairo, Egypt). Cells were kept in DMEM media with 100 mg/mL of streptomycin, 100 units/mL of penicillin and 10% of heat-inactivated fetal bovine serum in humidified, 5% (*v*/*v*) CO_2_ atmosphere at 37 °C.

### 3.10. In Vitro Cytotoxicity

Effect of the alcoholic extract and the isolated compounds on cell viability was evaluated to assess their safety utilizing SRB assay [53]. An aliquot of 100 µL cell suspension (5 × 10^3^ cells) was incubated with 100 μL media containing the test samples at concentrations (0.01, 0.1, 1, 10, 100 µg/mL) for 72 h. Media was removed and 150 μL of 10% TCA was added for fixing the cells that was further incubated at 4 °C for 1h. Cells were then washed with distilled water and 70 μL 0.4% SRB solution was added, and plates were placed in dark for 10 min. Plates were then washed with 1% acetic acid and left to dry overnight. 150 μL of 10 mM TRIS was then added and the absorbance was measured at 540 nm using a BMG LABTECH^®^-FLUOstar Omega microplate reader (Ortenberg, Germany). Experiments were done in triplicates and the IC_50_ was calculated.

### 3.11. Scratch Wound Healing Assay

The wound healing assay was conducted to evaluate the effect of different concentrations of the polar fraction and isolated compounds from *Artemisia Annua* on the migration ability of HSF cell line using the scratch wound healing assay as previously described [54]. In a coated 12-well plates, cells were seeded at density of 2 × 10^5^/well in 5% FBS-DMEM and cultured overnight at 37 °C and 5% CO_2_. On the following day, horizontal scratches were made into the confluent monolayer and the plates were washed with PBS and treated with fresh media containing the combined polar extract and the isolated compounds in concentrations of 10, 100, 1000 µg/mL. Control wells were done using only plain medium. The plates were incubated at 37 °C and 5% CO_2_ and images were captured using an inverted microscope at 24, 48 and 72 h time intervals. The acquired images were analyzed by MII ImageView software version 3.7. Experiments were performed in triplicates. Wound width was calculated as the average distance between the edges of the scratches. As cell migration enhanced, the wound width diminishes.

## 4. Conclusions

Results of this study introduced different mechanisms of action behind the antidiabetic effect of the combined polar fraction of the leaves of *A. annua* (ART-CQ), as well as three major dicaffeoylquinic acid derivatives contributing to the activity of this biologically active fraction. The dicaffeoylquinic acid derivatives were capable of inhibiting the activities of the DPPIVI, α-amylase and α-glucosidase enzymes in vitro. These actions could, at least partially, account for the well-recognized antidiabetic activity of *Artemisia annua* by increasing glucose-dependent insulin release, protecting pancreatic β-cells and delaying glucose absorption, thus reducing postprandial glucose excursion. Moreover, these compounds exhibited potential effectiveness in preventing diabetes complications via inhibiting aldose reductase enzyme and alleviating oxidative stress. Interestingly, some of these compounds promoted wound healing. Our findings gave new insights into the mechanisms of the antidiabetic effect of *A. annua* and its bioactive compounds that could represent promising lead compounds for the management of diabetes. However, additional future studies are needed to investigate other mechanisms of action of the antidiabetic effect of *A. annua* and these active metabolites and to exploit their efficacy in vivo as well.

## Data Availability

The data presented in this study are available in the article.

## Sample Availability

Samples of the compounds **1**, **2** and **3** are available from the authors.

## Abbreviations

ABTS	2:2′-azino-bis (3-ethylbenzothiazoline-6-sulphonic acid)
ART-CQ	Polar fraction of *Artemisia annua*
CNPG3	2-chloro-4-nitrophenyl -α-D-maltotrioside
Cpd	Compound
DPPIV	Dipeptidyl peptidase IV
DPPH	2, 2-Diphenyl-1-picrylhydrazil
FRAP	Ferric reducing antioxidant power
HSF	Human Skin Fibroblast
IC_50_	Inhibitory concentration 50
